# Quality of Maternal Death Documentation in Afghanistan: A Retrospective Health Facility Record Review

**DOI:** 10.3389/fgwh.2021.610578

**Published:** 2021-03-17

**Authors:** Farzana Maruf, Hannah Tappis, Jelle Stekelenburg, Thomas van den Akker

**Affiliations:** ^1^Global Financing Facility, World Bank Group, Kabul, Afghanistan; ^2^Faculty of Science, Athena Institute, Vrije Universiteit Amsterdam, Amsterdam, Netherlands; ^3^Jhpiego, Baltimore, MD, United States; ^4^Global Health Unit, Department of Health Sciences, University Medical Centre Groningen/University of Groningen, Groningen, Netherlands; ^5^Department of Obstetrics and Gynaecology, Leeuwarden Medical Centre, Leeuwarden, Netherlands; ^6^Department of Obstetrics and Gynaecology, Leiden University Medical Centre, Leiden, Netherlands

**Keywords:** maternal health, maternal mortality, pregnancy related death, medical records, Afghanistan, quality of documentation

## Abstract

**Objectives:** To assess the quality of health facility documentation related to maternal deaths at health facilities in Afghanistan.

**Methods:** Analysis of a subset of findings from the *2016 National Maternal and Newborn Health Quality of Care Assessment* in Afghanistan. At each facility, maternity registers were reviewed to obtain data related to maternity caseload, and number and causes of maternal deaths in the year preceding the survey. Detailed chart reviews were conducted for up to three maternal deaths per facility. Analyses included completeness of charts, quality of documentation, and cause of death using WHO application of International Statistical Classification of Disease to deaths during pregnancy, childbirth and the puerperium.

**Key findings:** Only 129/226 (57%) of facilities had mortality registers available for review on the day of assessment and 41/226 (18%) had charts documenting maternal deaths during the previous year. We reviewed 68 maternal death cases from the 41 facilities. Cause of death was not recorded in nearly half of maternal death cases reviewed. Information regarding mode of birth was missing in over half of the charts, and one third did not capture gestational age at time of death. Hypertensive disorders of pregnancy and obstetric hemorrhage were the most common direct causes of death, followed by maternal sepsis and unanticipated complications of clinical management including anesthesia-related complications. Documented indirect causes of maternal deaths were anemia, cardiac arrest, kidney and hepatic failure. Charts revealed at least eight maternal deaths from indirect causes that were not captured in register books, indicating omission or misclassification of registered deaths.

**Conclusion:** Considerable gaps in quality of recordkeeping exist in Afghanistan, including underreporting, misclassification and incompleteness. This hampers efforts to improve quality of maternal and newborn health data and priority setting.

## Introduction

The World Health Organization defines maternal death as the death of a woman while pregnant or within 42 days of termination of pregnancy, irrespective of the duration and site of the pregnancy, from any cause related to or aggravated by the pregnancy or its management but not from accidental or incidental causes (1). Maternal death is perceived as an indicator of quality of care. Reduction of such deaths has been on the global and national agendas for all countries for decades (2, 3).

Maternal mortality in Afghanistan has significantly declined from 1,600 deaths per 100,000 live births in 2002 (4) to 638 deaths per 100,000 live births in 2017 (5). This reduction is largely attributed to improvements in the health system and an increase in skilled birth attendance from 11% in 2003 to 58.8% in 2018 (6). However, since 2010, there has been a steady increase in violence and instability in Afghanistan (7). This has had adverse impacts on all aspects of the health system, resulting in closure of some health facilities, and increasing limitations on travel to facilities, especially by women and children (3, 8). Although there is no direct evidence to support this, it is plausible that instability may have also affected quality of record keeping and maternal death registration, thereby affecting reliability of maternal mortality figures.

Despite these challenges, the Ministry of Public Health (MoPH) in Afghanistan remains committed to reducing preventable maternal mortality and has set a national target for mortality reduction (3), considering the importance of the sustainable development goal (SDG) target for 2030, which is a reduction to <70 per 100,000 live births (9). In 2017, the lifetime risk of maternal mortality in Afghanistan was approximately 1 in 33 compared to the global lifetime risk of 1 in 190 (10). Main causes of maternal deaths in Afghanistan are hemorrhage, sepsis and complications of unsafe abortion. The high maternal mortality ratio in Afghanistan despite an increasing proportion of women giving birth in health facilities raises concerns regarding quality of maternity care at health facilities (11, 12).

Maternal death surveillance and response (MDSR) is widely implemented in low- and middle- income countries, as one strategy to identify and address health system weaknesses contributing to maternal deaths (13). In Afghanistan, the MoPH recognizes that regular audit of maternal deaths could contribute toward a better understanding of causes of death and stimulate a culture of learning, resulting in improved evidence-based decision making and practices at all levels of the health system (14).

However, while national guidelines for maternal and neonatal death surveillance and response exist, authorities acknowledge that maternal death audits have been poorly performed in Afghanistan and gaps exist in the quality of reported data (14, 15). Beyond statements in high level policy documents and health information system assessments, there is only limited information on quality of health facility documentation related to maternal death.

Previous studies in Afghanistan have assessed accuracy and validity of maternity care records and quality of cesarean section-related documentation, but none have examined the availability and completeness of documentation of maternal deaths (16, 17). We assessed the quality of health facility documentation related to maternal death in Afghanistan and implications for evidence-based decision making to improve maternal health outcomes.

## Methods

### Study Design

This retrospective health facility record review is a subset of Afghanistan's *National Maternal and Newborn Health Quality of Care Assessment*, a cross-sectional health facility assessment conducted in 2016. The assessment was designed to examine health facility readiness for basic and comprehensive emergency maternal and newborn care, and assess quality of routine antenatal care, childbirth and postpartum care, and management of selected obstetric and newborn complications (12, 18–20).

### Study Sample

The *National Maternal and Newborn Health Quality of Care Assessment* covered a total of 286 health facilities across all 34 provinces of Afghanistan. This included a census of all accessible public hospitals with an average of five births per day reported in the national health management information system in 2015 (*n* = 77), and a random sample of facilities with fewer than five births per day (*n* = 149). The sample size for low-volume facilities was calculated to provide a representative sample of the 1,736 public facilities with < five births per day, with a 10% margin of error, 5% alpha error, design effect of 1.5 due to stratification of facility types, and 5% oversampling for potential inaccessibility due to geography or insecurity. In addition, 20 private health facilities were purposively selected to provide a snapshot of care at private sector facilities in all regions of the country.

### Data Collection

Data collection was conducted by 32 female doctors and midwives trained on assessment objectives, data collection techniques and research ethics. Data collection methods for this paper included use of a facility inventory and record review tool to verify caseload, availability of relevant data and guidelines, as well as review of patient's folders or mortality charts. Data collection at health facilities with an average of at least five births per day was completed between May 14th, and August 3rd, 2016. Data collection at health facilities that averaged fewer than five births per day was completed from November 5, 2016, to January 5, 2017. At each facility, maternity registers were reviewed for information on the maternity caseload and number and causes of deaths in the year preceding the survey. Detailed chart reviews were conducted for up to three maternal deaths per facility; charts were requested for the three most recent maternal deaths in the previous year. For each chart, data collectors extracted information on client characteristics, location and mode of childbirth, timing and causes of death, and newborn outcomes. Data collectors reviewed charts for any information on factors contributing to maternal death and noted if women experienced any of the following: delayed arrival to health facility, delayed transfer to appropriate level of care, delay in care due to lack of supplies, delay in care due to absence or non-attendance on part of the health professional, delay in correct diagnosis (21). All data extraction was completed using a standardized form developed on the CommCare electronic data collection software platform.

### Data Analysis

Data was exported from CommCare to Excel for cleaning and analysis. We used visual inspection and descriptive statistics methods to analyze quality of documentation and completeness of charts, and case review methods to classify causes of death using WHO Application of ICD-10 to deaths during pregnancy, childbirth and the puerperium (ICD-MM coding). Facility types were defined as follows: (1) Specialized, Regional, and Provincial Hospitals; (2) District Hospitals (DHs) with an average of five or more births per day; (3) DHs and Comprehensive Health Centers (CHC) with an average of fewer than five births per day; and (4) Basic Health Centers (BHC), Sub Health Centers (SHC), Family Health Houses (FHH), and other primary health care facilities.

### Ethical Considerations

The 2016 Afghanistan National Maternal and Newborn Health Quality of Care Assessment protocol was approved by the ethical review boards of the Afghanistan Ministry of Public Health (361533) and the Johns Hopkins Bloomberg School of Public Health in Baltimore, Maryland (6,799). Written permission for data collection was also obtained from the in-charge of each facility.

## Results

Review of maternity registers showed an average number of 194 births per month at public facilities and 67 births per month at private facilities in the Afghan calendar year preceding the assessment (April 2015 – March 2016). An average of 175 antenatal care visits per month were reported at public and 107 antenatal care visits per month at private facilities (Table 1). Of public facilities, 149/226 (65.8%) reported providing 24-h coverage for service delivery compared to 18/20 (90%) private facilities. Only 129/226 (58%) of public facilities and 11/20 (55%) of private facilities had any data on maternal deaths recorded in standard facility registers. Overall, 207/226 (92%) public and 203/226 (90%) of private facilities had standard birth registers available for review on the day of assessment.

**Table 1:** Health facility characteristics (maternity caseload and data availability).

	**Public Facility Type**					
	**Specialized Hospital/Regional Hospital/Provincial Hospital (*n*=37)**	**District Hospitals with 5 or more deliveries per day (*n* = 40)**	**District Hospitals and Comprehensive Health Centers with 0-4 deliveries per day (*n* = 37)**	**Basic Health Center, Subhealth Center, Family Health House (*n* = 112)**	**ALL PUBLIC SECTOR (*n*=226)**	**Private Facilities (*n*=20)**
Number of antenatal care visits per month [average caseload, all visits (min, max)]	366 (0, 2,470)	349 (0, 1,485)	65 (0, 218)	80 (0, 2,470)	175 (0, 2,470)	107 (0, 430)
Number of deliveries per month [average (min, max)]	729 (136, 2,158)	301 (142, 1,233)	13 (0, 63)	15 (1, 98)	194 (0, 2,158)	67 (8, 218)
Number of postnatal visits per month (average [min, max])	292 (0, 2,115)	242 (0, 614)	31 (0, 145)	35 (0, 270)	111 (0, 2,115)	48 (0, 173)
% of health facilities providing 24-h coverage for delivery of services	100% (37)	100% (40)	57% (21)	44% (49)	66% (149)	90% (18)
Percent of facilities with maternity register available for review	97% (36)	100% (40)	89% (33)	88% (98)	92% (207)	90% (18)
*Average number of months with number of births recorded in maternity register in past year*	11	11	10	9	10	11
Percent of facilities with obstetric referral-in register available for review	65% (24)	60% (24)	38% (14)	40% (45)	47% (107)	35% (7)
*Average number of months with number of obstetric referrals-in recorded in register in past year*	7	7	4	5	6	4
Percent of facilities with obstetric referral-out register available for review	70% (26)	95% (38)	54% (20)	51% (57)	62% (141)	50% (10)
*Average number of months with number of obstetric referrals-out recorded in register in past year*	7	10	4	4	6	5
Percent of facilities with mortality or maternal death register available for review	65% (24)	70% (28)	43% (16)	54% (60)	57% (128)	55% (11)
*Average number of months with number of maternal deaths recorded in past year*	8	8	5	6	7	7

Specialized, Regional, and Provincial Hospitals which reported an average of more than 8500 births per year (Table 1), recorded an average of 18 maternal deaths per year, for an institutional maternal mortality ratio of 212 deaths per 100,000 births. High-volume DH, which reported an average of 3,600 births per year, recorded an average of 6 maternal deaths per year, for an institutional maternal mortality ratio of 167 deaths per 100,000 births. All maternal deaths recorded were attributed to direct causes. Specialized, Regional, and Provincial Hospitals reporting deaths saw an average of seven deaths due to complications of abortion (range 0–9), 6 deaths due to hemorrhage (range 0–7), and 2 deaths due to retained placenta (range 0–4) per year. High-volume district hospitals saw an average of 1 death due to complications of abortion (rang 0–1), two deaths due to hemorrhage (range 0–2) and two deaths due to retained placenta (range 0–2) per year. In the registers there were no deaths recorded due to indirect causes.

We reviewed patient charts for 68 women from 41 facilities (26 cases from Specialized, Regional, and Provincial Hospitals; 14 cases from DHs or CHCs, and one case from a private hospital), who died from direct or indirect causes related to the pregnancy, childbirth or clinical management. Seventeen charts did not record the age of the woman who died and 21 did not indicate gestational age. In more than half (39) mode of birth was not recorded. Seventeen did not record timing of death in relation to childbirth (during pregnancy, intrapartum or postpartum). Approximately half of the charts reviewed (35 of 68) recorded cause of death (Table 2).

**Table 2:** Maternal mortality case characteristics recorded in selected charts.

**Characteristics**	**Number of charts (*n*=68)**
**Age of women**	
Not recorded	17 (25%)
<20 years	4 (6%)
20–35 years	31 (47%)
>35 years	16 (24%)
**Type of delivery**	
Not recorded	39 (57%)
Vaginal delivery	17 (25%)
Assisted delivery	1 (1%)
Cesarean section	11 (16%)
**Gestational age**	
Not recorded	21 (31%)
<20 weeks	0 (0%)
20-40 weeks	44 (65%)
>40 weeks	3 (4%)
**Location of delivery**	
Not recorded	16 (24%)
Home birth	4 (6%)
Public health center (Comprehensive Health Center, Basic Health Center, Subhealth Center	5 (7%)
Public hospital (specialized Hospital, Regional Hospital, Provincial Hospital, District Hospital)	38 (56%)
On the way to health facility	2 (3%)
Private hospital	3 (4%)
**Women referred from**	
Not recorded	24 (35%)
Community health worker (Health Post)	1 (1.5%)
Health center (Comprehensive Health Center, Basic Health Center, Subhealth Center)	4 (6%)
Public hospital	6 (9%)
Private hospital	3 (4%)
Not referred	30 (44%)
**Timing of death**	
Not recorded	17 (25%)
During pregnancy	5 (7%)
Intrapartum (cesarean section)	7 (10%)
Intrapartum (vaginal delivery)	6 (9%)
Postpartum (cesarean section)	12 (18%)
Postpartum (vaginal delivery)	21 (31%)

Of the 51 charts with the woman's age recorded, four were <20 years old, 31 were between the age of 20 and 35 years and 16 were over 35 years of age. Of the 52 charts with birth location recorded, most (46) were facility births, whilst four women gave birth at home, and two women on the way to the health facility. Mode of birth was only recorded for 29 cases: 18 women gave birth vaginally and 11 by cesarean section. Fourteen women were referred from lower level health facilities and private sector to higher levels of care. Of the 51 charts with timing of death recorded, 33 were deaths occurring postpartum, 13 intrapartum and 5 antepartum.

**Figure 1:**
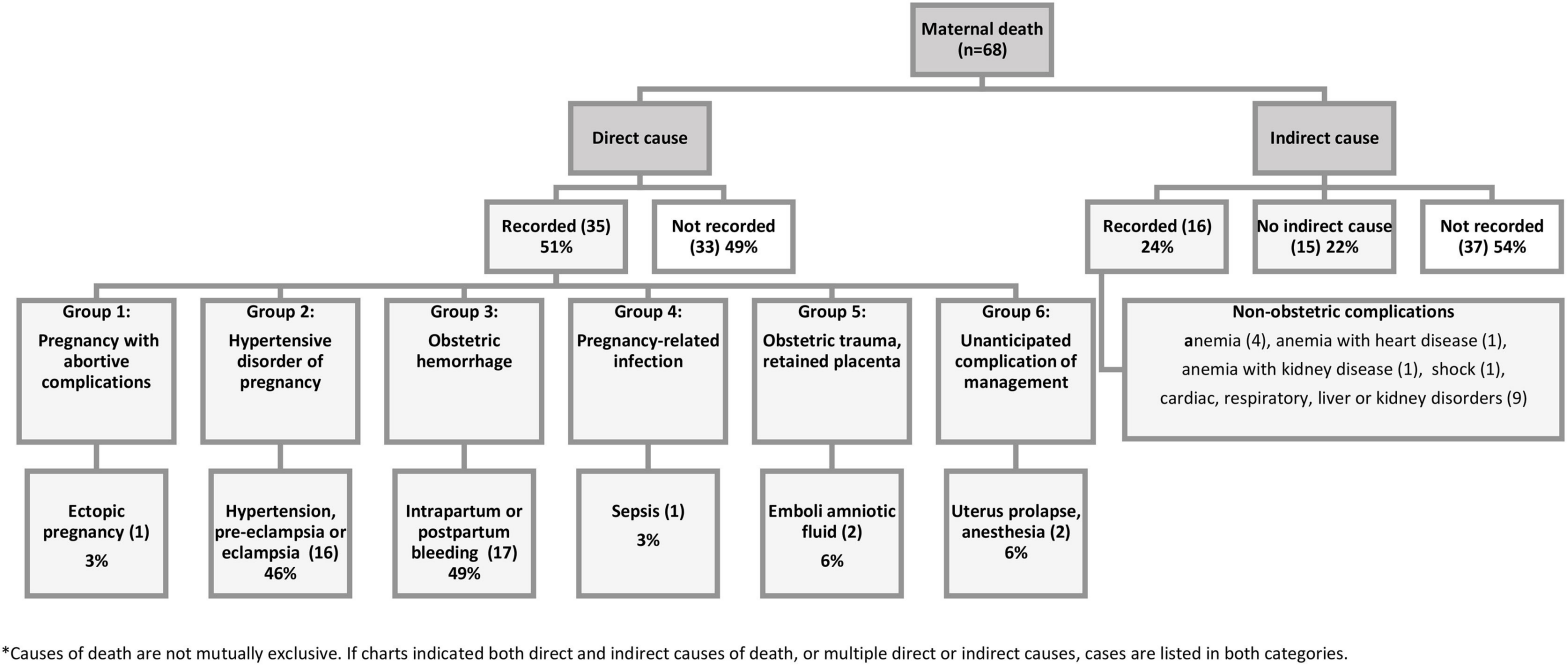
Causes of maternal death recorded in selected charts*.

The review of patient's charts showed that the main causes of mortality were obstetric hemorrhage (17), hypertensive disorder, preeclampsia and eclampsia (16), amniotic fluid embolus (2), unanticipated complication of management such as anesthesia (2), sepsis (1), and ectopic pregnancy (1). Fifteen charts indicated no indirect causes of maternal death, while 37 did not record any information on indirect causes. The causes of death listed in 16 cases reporting indirect causes were cardiac, respiratory, liver and kidney disorders (9), anemia (4) and anemia with heart and kidney disease (2), and shock (1) (Figure 1). Sixteen charts included multiple causes for maternal deaths.

**Figure 2:**
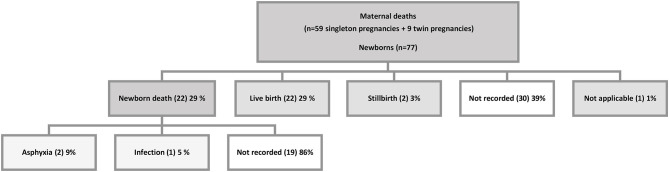
Newborn outcome recorded in selected maternal mortality charts.

The 68 maternal mortality charts reviewed included 59 singleton pregnancies and 9 twins (Figure 2). Of the 77 pregnancies, birth outcomes were only reported for 47. Newborn outcomes were not recorded in 30 cases; 22 newborns were reported alive. Twenty-two were recorded early newborn death, two stillbirths, and one was an ectopic pregnancy in early pregnancy. Causes of death were only recorded for three early newborn deaths: asphyxia (2) and infection (1).

Fifty-five of the 68 maternal mortality charts reviewed included information on factors contributing to maternal deaths (Table 3). Thirty-seven charts included factors contributing to delays in receiving quality care (delayed referral from other facilities, delay getting timely treatment, inadequate skills of providers or delay in correct diagnosis, lack of supplies, medicine and blood, and absence or delay in attendance of human resources), 21 charts included information on delays to reach a health facility and 2 charts reported poor antenatal care as a factor contributing to maternal mortality. In 13 charts, such reasons were not recorded.

**Table 3:** Recorded factors contributing to maternal death^*^.

	**Number of charts (*n* = 68)**
Delayed arrival to facility	21 (31%)
Delayed transfer to appropriate level of care	5 (7%)
Didn't receive any treatment before referral from one health facility to another	1 (2%)
Delay due to lack of supplies and medicine	13 (19%)
Lack of blood	1 (2%)
Delay due to absence or slowness of human resources	11 (16%)
Delay in correct diagnosis	6 (9%)
Lack of antenatal care	2 (3%)
No contributing factors recorded	13 (19%)

* *The contributing factors are not mutually exclusive*.

## Discussion

Our study shows remarkable weaknesses in quality of documentation and record keeping, at both public and private facilities in Afghanistan. We found glaring gaps in completeness of recordkeeping. Critical information on characteristics of deceased were missing in nearly half of the records. Our results are in line with other studies from Afghanistan that have indicated that quality of medical record keeping is extremely low (16, 17, 22). Concerns that clients' records do not accurately reflect the true clinical picture have been documented, specifically at large hospitals (Specialized, Regional, and Provincial Hospitals) due to resource constraints, work overload, and apprehension of personal and political accountability for maternal death (14, 16). In addition, a study in Ethiopia denoted that mere forgetfulness in facilities with high numbers of deaths, limited knowledge of the reporting procedures and unavailability of specific guidelines may lead to serious underreporting of maternal deaths (23, 24).

Charts reviewed suggest that the most commonly reported direct causes of maternal deaths were obstetric hemorrhage, eclampsia, and abortions; fewer cases were recorded on maternal sepsis and complications of anesthesia. Hence, urgent effort is needed to first address these immediate causes of death for many women, while putting in longer-term efforts to strengthen the health system and working on social determinants that lie outside the realm of the health sector, in a multifaced approach (2, 3). As per the chart review, the indirect causes of maternal deaths seen at health facilities in Afghanistan anemia, cardiac arrest, kidney and hepatic failure, and shock. Presumably, some of these indirect causes (chronic anemia and shock) were either a contributing factor or a result of direct causes of maternal deaths. However, no single indirect cause of maternal death was recorded in the facility register. We found notable discrepancies between reported causes of maternal death in facility registers and patients' charts. The charts reviewed showed at least eight maternal deaths due to indirect causes that should have been recorded in the registers, indicating either omission or misclassification of deaths in the register, or both.

Accurate classification of causes of maternal mortality is vital for decision making and provision of care; otherwise there could be misclassification and under-identification of maternal deaths at facilities, where a woman of reproductive age may die in a medical or surgical ward with an undiagnosed obstetric complication (25). For programmatic purposes, indirect causes of maternal mortality require greater attention, not just for purposes of reporting, but also for timely decision making and interventions to prevent these deaths (26).

Notably, reliability of facility level data will become even more important when frontline clinicians accurately record clinical activities and related outcomes in charts. Information on the medical records can be used to improve health service quality (16, 27). Moreover, these records are used for the surveillance of maternity care and outcomes, in addition to monitoring and evaluation of progress. Having reliable data for action and accountability at subnational levels will provide better opportunities for equity-based interventions, optimal decision making and advantages for women who seek care in the formal health facilities (28, 29).

The private health sector in Afghanistan is dynamic, rapidly expanding, and largely unregulated (30). Very few private health facilities report into national health management information system or keep reliable records of services, leaving great uncertainty on the proportion of maternal deaths that may be occurring at these facilities. The purposive snapshot of private health facilities included in this assessment confirms that maternal deaths are occurring at private health facilities, and more attention to quality of documentation and reporting practices at both public and private facilities are needed to fully understand the state of maternal health care services in Afghanistan.

In Afghanistan, the poor quality of medical records, lack of knowledge and skills, staff turnover, lack of coordination, the culture of blame and inadequate resources are barriers to conducting effective death reviews (14, 31, 32). Effective maternal death reviews require supportive political and legal supports, individual commitment, and an environment conducive to learning as integral part of improving service coverage and quality of care. The Afghan health system needs strengthening in areas related to strategies and resources on notification of births and deaths, and medical certification of causes of death, and infrastructure to record the causes of death in the medical records (14).

Providing targeted refresher training on clinical record keeping, improved compliance by health care providers, staff support and fostering a culture of secrecy and professional protectionism, and non-punitive responses on medical errors, and strong leadership at national level and at lower levels of governance will lead to accuracy, reliability and completeness of medical records (16, 22, 31). A recent study suggests that periodic data audits were successful in improving data quality and use (33).

There are some examples of successful large-scale implementation of MDSR in low- and middle-income countries, including in Malaysia, Sri Lanka and India. Evidence suggests that such implementation requires considerable efforts to identify and address maternal deaths and associated factors (13). In countries with an established MDSR system, for example Malaysia, routine notification of deaths from facilities and upgrading the functional vital registration system to include a tick box for maternal deaths on death certificates enables more effective identification of all maternal deaths (34). In countries where vital registration systems are weak, skilled birth attendance rates are low, and many deaths occur without any contact with health facilities; like Afghanistan, identification and notification of maternal deaths at community level is an important source of information that may reduce underreporting.

MDSR remains a vertical intervention in Afghanistan, operated in parallel to the country's disease surveillance system. A study in Ethiopia has emerged the importance of MDSR embedment within the existing disease surveillance system as a routine practice rather than a stand-alone system, with a greater supervisory support to improve the reporting and response mechanism (24).

There are considerable methodological shortcomings of surveys to measure maternal mortality in Afghanistan, exacerbated by the fragile context. Efforts should be redirected to improve access to and quality of routine maternal health data to obtain real time-insights, with increased longer-term investment in civil registration and vital statistics (22).

The use of electronic medical records (EMR) in Cameroon and Malawi has shown an increase in completeness and accuracy of medical records (35, 36). A study in Ghana has acknowledged that lack of financial and human resources would limit EMR implementation and sustainability (35). The government of Afghanistan plans to move toward the ambitious agenda of digitalization of medical records to improve quality and accessibility of timely information (37). There is need for a comprehensive study to inform facilitators and barriers with regards to introduction and scale up of EMR in Afghanistan.

Our study is not without limitations. It was designed to assess the quality of broader maternal and newborn health services. Therefore, only three most recent patients' charts per facility were reviewed, not all deaths in the previous year, so data on case characteristics and causes of death cannot be considered representative of all maternal deaths in Afghan health facilities. The private-sector snapshot provided only a limited picture of maternal mortality in private health facilities in Afghanistan and cannot be compared with nationally representative data from public health facilities. Despite these limitations, our study shines a light on the deplorable quality of record keeping and documentation of maternal health outcomes, and highlights challenges in relying on facility records for estimations of maternal mortality.

## Conclusion

Our study identifies considerable concerns with regard to the quality of record keeping in Afghanistan. These encompass under reporting, misclassification, and incomplete documentation. Improvements in record-keeping and health management information systems are critical to improve maternal and newborn health outcomes. Improving the quality of maternal and newborn health data requires robust political support and investment at various levels to support real-time clinical and political decision making and priority setting. This paper serves as a call to improve documentation and analysis of causes of maternal deaths amidst insecurity in Afghanistan, and to systematically improve accountability for coordinated, data-driven efforts to address factors associated with preventable maternal mortality.

## Data Availability Statement

Data is available from the principal investigator or upon reasonable request or signature of a data use agreement.

## Ethics Statement

The studies involving human participants were reviewed and approved by the ethical review boards of the Afghanistan Ministry of Public Health (361533) and the Johns Hopkins Bloomberg School of Public Health in Baltimore, Maryland (6799).

## Author Contributions

FM served as co-trainer for the 2016 Afghanistan National Maternal and Newborn Health Quality of Care Assessment, wrote the first draft of this manuscript, led data analysis, interpretation and revision of this article. TvdA contributed to the writing and revision of the manuscript. JS served as a study advisory board member and contributed to the review and revision of the manuscript. HT served as principal investigator, led the data analysis, and contributed to the interpretation of study findings and revision of the manuscript. All authors read and approved the final manuscript.

## Conflict of Interest

The authors declare that the research was conducted in the absence of any commercial or financial relationships that could be construed as a potential conflict of interest.

## Abbreviations

BHC: basic health center
CHC: comprehensive health center
DH: district hospital
FHH: family health house
MDSR: maternal death surveillance system
MoPH: Ministry of Public Health
SBA: skilled birth attendant
SDG: sustainable development goal
SHC: sub-health center
WHO: World Health Organization.
